# Comprehensive Analysis of Glutamate-Rich WD Repeat-Containing Protein 1 and Its Potential Clinical Significance for Pancancer

**DOI:** 10.1155/2021/8201377

**Published:** 2021-09-27

**Authors:** Yumeng Wu, Xuming Wu, Yuanyuan Li, Wenjing Zhao, Yanping Yue, Biao Wu, Jibin Liu, Xudong Chen, Aiguo Shen

**Affiliations:** ^1^Cancer Research Center Nantong, Affiliated Tumor Hospital of Nantong University, China; ^2^Nantong Fourth People's Hospital, China; ^3^Department of Pathology, Affiliated Tumor Hospital of Nantong University, China

## Abstract

**Methods:**

The expression level of GRWD1 in human cancer tissues was analyzed using the Tumor Immune Evaluation Resource (ver. 2.0, TIMER2), Gene Expression Profiling Interactive Analysis (ver. 2, GEPIA2), and UALCAN databases. The Kaplan-Meier plotter was utilized to analyze the survival data. Spearman's correlation analysis was used to find out the correlation between the expression level of GRWD1 and predictive biomarkers, such as tumor mutation burden (TMB) and microsatellite instability (MSI). Furthermore, the MEXPRESS website was used to study the potential relationship between DNA methylation level of GRWD1 and pathological staging. We utilized the “immune” module provided on the TIMER2 website to explore the relationship between the expression level of GRWD1 and immune infiltration in all types of cancer in TCGA. Pearson's correlation analysis was used to investigate the correlation between the expression level of GRWD1 and the expression levels of immune checkpoint-related genes. For protein expression analysis, we used the CPTAC module provided by the UALCAN portal to compare the total protein and phosphorylated protein level of GRWD1 in adjacent normal and tumor tissues.

**Results:**

GRWD1 was overexpressed in tissues of most types of cancer, in which the expression levels of GRWD1 in the kidney chromophobe (KICH), kidney renal papillary cell carcinoma (KIRP), and kidney renal clear cell carcinoma (KIRC) tissues showed an opposite trend, and the expression level of GRWD1 was correlated to only the KIRC tumor stage. The results of survival analysis showed that the expression level of GRWD1 was significantly associated with overall survival in six types of cancer and disease-free survival (DFS) in three types of cancer. Importantly, the increased expression level of GRWD1 was strongly correlated with prognosis of KIRC patients. There was a positive relationship between the expression level of GRWD1 and immune cell infiltration in several types of cancer, and the expression level of GRWD1 was also positively correlated with TMB, MSI, and DNA methylation in some types of cancer. The results of Kyoto Encyclopedia of Genes and Genomes (KEGG) pathway enrichment analysis revealed that “ubiquitin mediated proteolysis,” “spliceosome,” and “nucleotide excision repair” were involved in the effect of GRWD1 expression on tumor pathogenesis.

**Conclusion:**

This pancancer analysis provided a comprehensive overview of the carcinogenic effects of GRWD1 on a variety of human cancers. The results of bioinformatics analysis indicated GRWD1 as a promising biomarker for detection, prognosis, and therapeutic assessment of diverse types of cancer, and GRWD1 could act as a tumor suppressor in KIRC.

## 1. Introduction

Cancer is the leading cause of death globally, as well as being an important obstacle to increasing life expectancy. The rates of cancer morbidity and mortality are universally increasing. According to the World Health Organization (WHO) estimates, in 2019, cancer was the first or second leading cause of death in 112 countries [1]. Pancancer analysis has been widely used in cancer research, which has revealed the common characteristics and heterogeneity of diverse types of cancer, facilitating the discovery of new targets for cancer treatment [2].

Glutamate-rich WD repeat-containing protein 1 (GRWD1), also known as WDR28, can interact with different proteins involved in transcription, translation, cell cycle progression, DNA replication and repair, chromatin organization, and ubiquitin-mediated proteolysis [3]. GRWD1 encodes a 446 amino acid protein, containing a glutamate-rich region, followed by four WD repeat sequences [4]. Nozomi et al. found that GRWD1 is a novel histone-binding protein, regulating chromatin dynamics and loading of the minichromosome maintenance (MCM) protein complex at replication origins [5, 6]. GRWD1 is also a negative regulator of P53. It physically and functionally interacts with ribosomal protein L11 (RPL11) and ribosomal protein L23 (RPL23) to promote the activity of murine double minute 2 (MDM2) protein on P53 [7–9].

Recent studies have confirmed that GRWD1 can directly interact with P53 and negatively regulate the transcriptional activity of P53 [10]. The previously described GRWD1-negative regulation of P53 plays a crucial role in low-grade gliomas (LGGs). The overexpression of GRWD1 is also associated with poor prognosis of patients with non-small cell lung cancer and colorectal cancer [3, 11, 12]. However, previous studies limited the evaluation of GRWD1 to few types of cancer, and its role in other types of cancer has still remained elusive.

In order to explore the expression level of GRWD1 in various types of cancer by pancancer analysis, the present study was conducted using bioinformatics tools, and it was found that GRWD1 was highly expressed in almost all types of cancer. The expression level of GRWD1 was significantly correlated with survival, immune cell infiltration, and the mutational status of tumors.

## 2. Materials and Methods

### 2.1. Gene Expression Analysis

We used the “Gene_DE” module in the TIMER2 website (http://timer.cistrome.org/) to identify the expression level of GRWD1 in adjacent normal and tumor tissues. For certain tumors without normal or with highly limited normal tissues (e.g., glioblastoma multiforme (GBM) and pancreatic adenocarcinoma (PAAD)), we used the “Box Plots” module in the Gene Expression Profiling Interactive Analysis, ver. 2 (GEPIA2) web server to obtain box plots of the expression difference between these tumor tissues and the corresponding normal tissues of the Genotype-Tissue Expression (GTEx) database, under the setting of *P* value (cutoff) equal to 0.01, log_2_FC (cutoff) equal to 1, and “Match TCGA normal and GTEx data.” We also obtained a violin map of GRWD1 expression in all TCGA tumors at different pathological stages (stage I, stage II, stage III, and stage IV) through the “Pathological Stage Plot” module in the GEPIA2 and applied log_2_ (TPM (transcripts per million) +1) transformed expression data to the violin chart. For protein expression analysis, we used the CPTAC module provided by the UALCAN portal (http://ualcan.path.uab.edu/analysis-prot.html) to compare the expression levels of total protein and phosphorylated protein of GRWD1 in adjacent normal and tumor tissues.

### 2.2. Survival Analysis

We utilized “survival map” module in the GEPIA2 to obtain the rates of overall survival (OS) and disease-free survival (DFS) in all types of cancer in TCGA. Cutoff-high (50%) and cutoff-low (50%) values were used as the expression thresholds for splitting the high-expression and low-expression cohorts [13]. The hypothesis test adopted the log-rank test, and the survival curves could be drawn using the “survival analysis” module in the GEPIA2.

### 2.3. Genetic Alteration Analysis

We used the cBioPortal website (https://www.cbioportal.org/) to find out the results of alteration frequency, mutation type, and copy number aberrations (CNAs) of GRWD1 among all types of cancer in TCGA. The “TCGA Pan Cancer Atlas Studies” in “Quick Selection” was selected, “Query by gene” was chosen, and “GRWD1” was typed to query genetic alteration characteristics of GRWD1, and the results were displayed in the “Cancer Type Summary” module. We also used the “Comparison/Survival” module to obtain the rates of OS, DFS, and progression-free survival (PFS) of uterine corpus endometrial carcinoma (UCEC) cases from the TCGA database with or without GRWD1 gene alteration. A Kaplan-Meier survival curve was plotted, and *P* values for the log-rank test were calculated.

### 2.4. Immune Infiltration Analysis

We utilized the “immune” module provided on the TIMER2 website to explore the relationship between the expression level of GRWD1 and immune infiltration in all types of cancer in TCGA. Cancer-related fibroblasts and endothelial cells in the “Gene” option were selected, and xCell, EPIC, MCPcounter, and TIDE algorithms were employed to find out the correlation between the expression level of GRWD1 and tumor immune infiltration. The *P* values and partial correlation (cor) values were obtained via the purity-adjusted Spearman's rank correlation test. The results were visualized as heat maps.

### 2.5. Immune Checkpoint Gene Analysis

A total of 33 human tumor RNA-seq data samples were downloaded from Genomic Data Commons (https://portal.gdc.cancer.gov/). To make reliable immune infiltration estimations, TIMER2 utilizes the “immunedeconv” package in the R programming language that integrates six state-of-the-art algorithms, including TIMER, xCell, MCPcounter, CIBERSORT, EPIC, and quanTIseq. The expression levels of 8 immune checkpoint-related genes (SIGLEC-15, IDO1, CD274, HAVCR2, PDCD1, CTLA4, LAG3, and PDCD1LG2) were detected, and the R 4.0.3 programming language was used to perform the statistical analysis.

### 2.6. GRWD1-Related Gene Enrichment Analysis

First, a single protein name “GRWD1” and an organism “Homo sapiens” were used to query in the BioGRID database (https://thebiogrid.org/). Subsequently, in the “Network” module, only physical interactions in the “FILTERS” were shown, and the “MINIMUM EVIDENCE” was set to 5. Afterward, the “Similar Genes Detection” module in the GEPIA2 was employed to obtain the top 100 GRWD1-related genes on tumor tissue samples from TCGA. The “correlation analysis” module in the GEPIA2 was utilized to perform Pearson's correlation analysis on GRWD1-related genes. The log_2_ TPM was applied for the dot plot. The *P* value and the correlation coefficient (*R*) were indicated.

Moreover, the “Gene_Corr” module in the TIMER2 was utilized to supply the heat map data of the selected genes, containing the partial correlations (cor) and *P* values obtained from the purity-adjusted Spearman's rank correlation test. The Jvenn website (http://jvenn.toulouse.inra.fr/app/example.html) was used to draw a Venn diagram to find GRWD1-related genes interacting with similar expression patterns. We combined these two datasets for conducting the Gene Ontology (GO) and Kyoto Encyclopedia of Genes and Genomes (KEGG) pathway enrichment analyses using the OmicShare tools (https://www.omicshare.com/tools).

## 3. Results

### 3.1. Gene Expression Analysis

First, we explored the carcinogenic effect of the human GRWD1 gene (mRNA is NM_031485.4, protein is NP_113673.3, Figure S1A). As shown in Figure S1B, the structure of the GRWD1 protein is conserved among different species (e.g., H. sapiens, M. musculus, P. troglodytes, and A. gambiae), and it is mainly composed of the WD40 (cl02567) domain and the CAF1C (pfam12265) domain. The phylogenetic tree data (Figure S2) indicated the evolutionary conservation of the GRWD1 protein among different species. In brief, the high degree of conservation among different species suggests that GRWD1 plays a vital role in the fundamental biological processes.

We then analyzed the expression levels of GRWD1 in different cells and nontumor tissues. As illustrated in Figure S3A, according to the combination of the Human Protein Atlas (HPA), GTEx, and Functional Annotation of Mammalian Genome 5 (FANTOM5) datasets, GRWD1 has the highest expression level in the pancreas, followed by the skeletal muscle and liver (Figure S3A). However, GRWD1 was expressed in all the tissues, demonstrating a low tissue-specific mRNA level. In the different types of cells in the HPA/Monaco/Schmiedel dataset, the GRWD1 expression also showed a low single cell-type specificity (Figure S3B).

**Figure 1:**
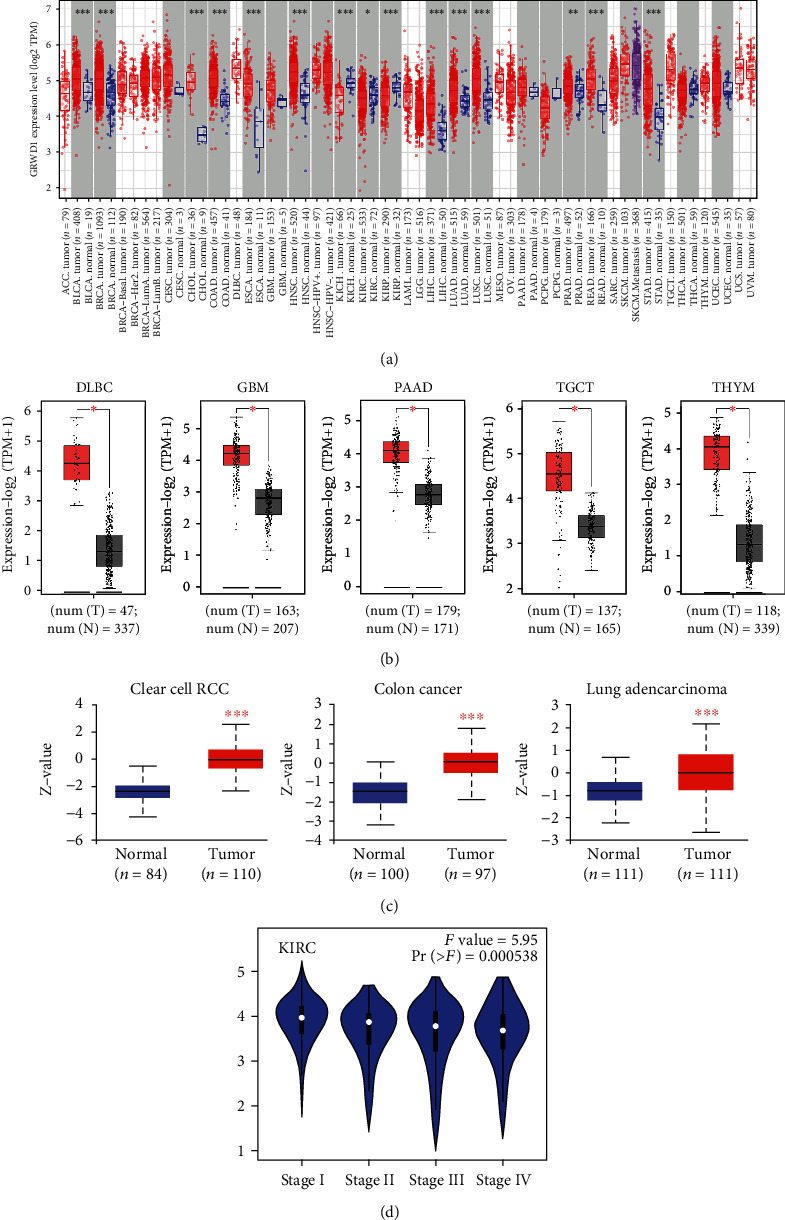
The expression levels of the GRWD1 gene in different types of cancer and pathological stages. (a) The expression levels of the GRWD1 in various types of cancer or subtypes of cancer. ^∗^*P* < 0.05; ^∗∗^*P* < 0.01; ^∗∗∗^*P* < 0.001. (b) The expression levels of GRWD1 in DLBC, GBM, PAAD, TGCT, and THYM tissues obtained from TCGA, while those levels in normal tissues found in the GTEx database were used as controls. Box plots of the expression difference between the tumor tissues and the corresponding normal tissues. ^∗^*P* < 0.01. (c) Comparison of total protein expression of GRWD1 in normal tissues and in ccRCC, colon cancer, and LUAD tissues. ^∗∗∗^*P* < 0.001. (d) According to the data obtained from TCGA, the expression level of the GRWD1 gene was analyzed according to the pathological stages of KIRC (Stages I, II, III, and IV). TPM values were also converted to logarithmic scale using log_2_ (TPM).

In the TCGA database, we used TIMER2 to investigate the differential expressions of GRWD1 in 33 tumor tissues. As depicted in Figure 1(a), the expression levels of GRWD1 increased in bladder urothelial carcinoma (BLCA), breast invasive carcinoma (BRCA), cholangiocarcinoma (CHOL), colon adenocarcinoma (COAD), esophageal carcinoma (ESCA), head and neck squamous cell carcinoma (HNSCC), liver hepatocellular carcinoma (LIHC), lung adenocarcinoma (LUAD), lung squamous cell carcinoma (LUSC), rectum adenocarcinoma (READ), stomach adenocarcinoma (STAD), and prostate adenocarcinoma (PRAD) (*P* < 0.01); however, in kidney chromophobe (KICH), kidney renal papillary cell carcinoma (KIRP) (*P* < 0.001), and kidney renal clear cell carcinoma (KIRC) (*P* < 0.05) tissues, the mRNA levels of GRWD1 in adjacent normal tissues were higher than those in tumor tissues. Using data collected from GTEx, normal tissues were used as controls, and we found that the expression levels of GRWD1 in diffuse large B-cell lymphoma (DLBC), glioblastoma multiforme (GBM), pancreatic adenocarcinoma (PAAD), testicular germ cell tumors (TGCTs), and thymoma (THYM) tissues were higher than those in adjacent normal tissues (Figure 1(b)). However, in other types of cancer, such as LGGs, ovarian serous cystadenocarcinoma (OSC), skin cutaneous melanoma (SKCM), uterine carcinosarcoma (UCS), uterine corpus endometrioid carcinoma (UCEC), acute myeloid leukemia (AML), cervical squamous cell carcinoma and endocervical adenocarcinoma (CESC), BRCA, and adrenocortical carcinoma (ACC), we did not detect significant differences in the expression levels of GRWD1 in adjacent normal and tumor tissues (Figure S4).

The CPTAC dataset was used to examine differences in GRWD1 protein levels in 7 tumor tissues, and it was revealed that GRWD1 protein levels were higher in clear cell renal cell carcinoma (ccRCC), colon cancer, and LUAD than in adjacent normal tissues (Figure 1(c), *P* < 0.01).

In addition, we used the “Pathological Staging Diagram” module in the GEPIA2 to figure out the correlation between the GRWD1 expression level and the pathological stage. It was found that the expression level of GRWD1 was only statistically significant in KIRC tissues (Figure 1(d), *P* < 0.05), rather than in other types of cancer (Figure S5).

### 3.2. Survival Analysis

**Figure 2:**
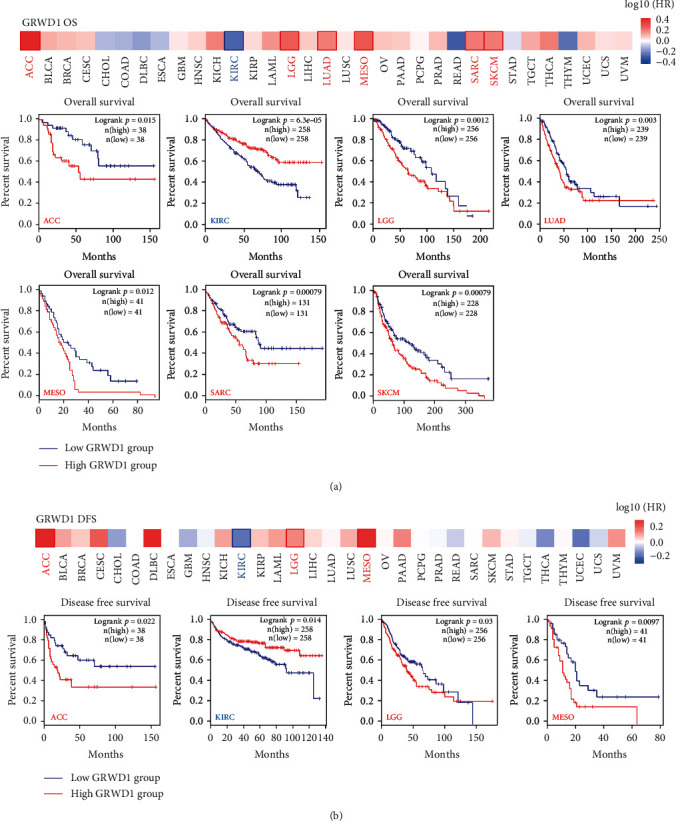
The relationship between the expression level of GRWD1 gene in TCGA and survival data. The expression level of GRWD1 and the rates of (a) overall survival and (b) disease-free survival in different tumor tissues in TCGA. The Kaplan-Meier survival curves were plotted.

We used the TCGA database to study the relationship between the expression level of GRWD1 and the survival of patients with different types of cancer. We divided cancer patients into high expression level and low expression level groups according to the expression level of GRWD1. In the TCGA database, the high expression levels of GRWD1 in ACC (*P* = 0.015), LGG (*P* = 0.0012), LUAD (*P* = 0.003), MESO (*P* = 0.012), SARC (*P* = 0.024), and SKCM (*P* = 0.00079) tissues were associated with a shorter OS, indicating a poor prognosis. As illustrated in Figure 2(b), in the TCGA database, the high expression levels of GRWD1 in ACC (*P* = 0.022), LGG (*P* = 0.03), and MESO (*P* = 0.0097) tissues were associated with a shorter DFS, demonstrating a poor prognosis. However, the low expression of GRWD1 in KIRC tissue was associated with shorter OS and DFS, confirming a poor prognosis (Figures 2(a) and 2(b); *P* < 0.001 and *P* = 0.014, respectively).

Furthermore, we used the Kaplan-Meier plotter (http://kmplot.com/analysis/index.php?p) to analyze the survival data, and we found that the high expression level of GRWD1 in breast cancer tissue was correlated to a longer recurrence-free survival (RFS) (Figure S6A, *P* < 0.001). As shown in Figure S6D, the high expression level of GRWD1 in lung cancer tissue was associated with a poor prognosis of patients with first progression (FP) (*P* = 0.0016), confirming shorter OS (*P* < 0.001) and postprogression survival (PPS) (*P* < 0.001). In addition, a low expression level of GRWD1 in ovarian cancer tissue indicated longer OS (*P* = 0.0075) and RFS (*P* = 0.039), while shorter PPS (*P* = 0.021) (Figure S6E). We failed to detect the correlation between the expression level of GRWD1 and prognostic indicators in liver cancer and gastric cancer tissues (Figures S6B, C; all *P* > 0.05). Overall, the expression level of GRWD1 varied in different types of cancer, confirming tumor heterogeneity.

### 3.3. Genetic Alteration Analysis

**Figure 3:**
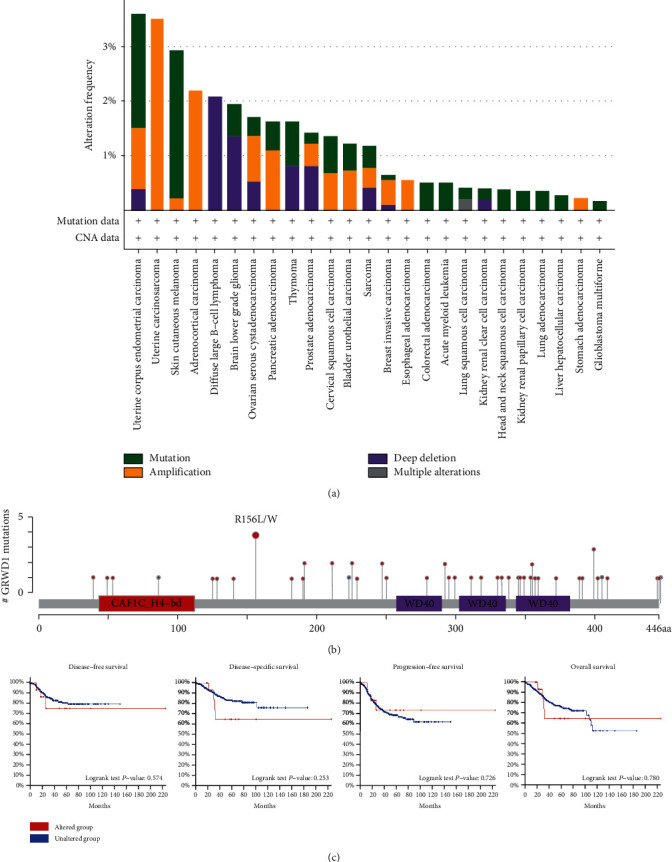
The genetic alterations of GRWD1 in different types of cancer in TCGA. We used the cBioPortal tool to analyze the genetic alterations of GRWD1 in different types of cancer in TCGA. (a) Alteration frequency of GRWD1 gene in UCEC patients. (b) Type, location, and number of cases with GRWD1 gene. (c) The potential correlation between mutational status and DFS, DSS, PFS, and OS of UCEC patients.

The cBioPortal presented the genetic alterations of GRWD1 in different tumor samples in the TCGA cohort. As depicted in Figure 3(a), the highest alteration frequency of GRWD1 (>3%) appeared in UCEC patients with the “mutation” type. Secondly, the alteration frequency of GRWD1 in UCS patients was the “amplification” type, and the alteration frequency was slightly higher than 3% (Figure 3(a)). It is noteworthy that all DLBC cases (alteration frequency was about 2%) had the copy number deletion of GRWD1 (Figure 3(a)). The type, location, and number of cases with GRWD1 gene are shown in Figure 3(b). The relationship between mutational status and DFS, DSS, PFS, and OS of UCEC patients is presented in Figure 3(c). GRWD1 mutations had no correlation with DFS, DSS, PFS, and OS, which might be due to the small sample size.

Further evidence showed that tumor mutation burden/microsatellite instability (TMB/MSI) can predict the response to immunotherapy [14]. We then analyzed the correlation between the expression level of GRWD1 and TMB/MSI in all tumor samples from TCGA. As displayed in Figure S7, the expression level of GRWD1 was positively correlated with TMB in ACC (*P* = 0.00086), LGG (*P* = 1.39*e* − 11), UCS (*P* = 0.031), SARC (0.00018), and READ (*P* = 0.024) tissues, while it was negatively correlated with TMB in pheochromocytoma and paraganglioma (PCPG; *P* = 0.0077) and THCA (*P* = 7.08*e* − 06) tissues. The expression level of GRWD1 was also positively correlated with the MSI in LIHC (*P* = 0.0022), uveal melanoma (UVM; *P* = 0.045), KIRC (*P* = 3.59*e* − 06), and SARC (*P* = 6.71*e* − 07) tissues, whereas it was negatively correlated with the MSI in READ tissue (*P* = 0.0022) (Figure S8). Previous researches showed that patients with a high TMB/MSI had a better response rate to immune checkpoint inhibitors [15–17], and this result is worthy of further study.

### 3.4. DNA Methylation Analysis

DNA methylation is an epigenetic modification that is correlated with gene repression, and it plays an important role in gene regulation, development, and tumorigenesis. We used the UALCAN website to find out the DNA methylation level of GRWD1 in cancer tissues that was significantly downregulated in TGCT (Figure S9A, *P* < 0.0001). Then, we used the MEXPRESS website to study the potential relationship between the DNA methylation level of the GRWD1 gene and pathological features of TGCT. In addition to a significant negative correlation between probes in the promoter region and the expression level of GRWD1, we also detected a significant negative correlation between the majority of probes in nonpromoter regions and the expression level of GRWD1. We also found that the DNA methylation level of GRWD1 was correlated with “histological type” and “history of an undescended testicle,” as illustrated in Figure S9.

### 3.5. Protein Phosphorylation Analysis

**Figure 4:**
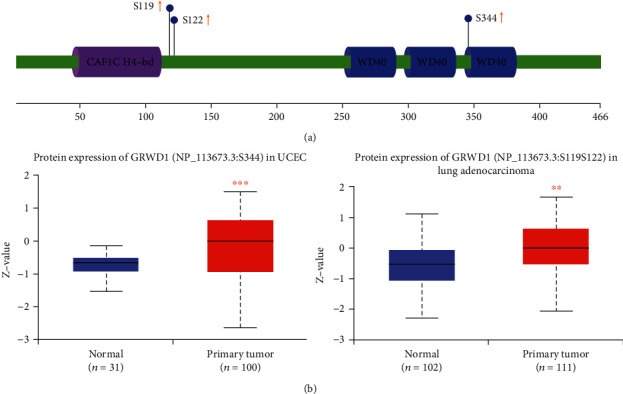
Phosphorylation analysis of GRWD1 protein in different tumor tissues. Using the CPTAC dataset, we compared the phosphorylation levels of GRWD1 (NP_113673.3, S119, S122, and S344 sites) in adjacent normal and tumor tissues via the UALCAN. (a) Positions of the phosphorylated amino acids. (b) Comparison of differences in the expressions of phosphorylated GRWD1 between adjacent normal and UCEC and LUAD tissues.

We utilized the CPTAC dataset to compare the phosphorylation levels of GRWD1 in adjacent normal and tumor tissues.  Figure 4(a) is a summary of phosphorylation sites with significant differences in the CPTAC dataset. It was revealed that the phosphorylation levels of GRWD1 in UCEC and LUAD tissues were higher than those in adjacent normal tissues (Figure 4(b), all *P* < 0.01).

### 3.6. Immune Infiltration Analysis

**Figure 5:**
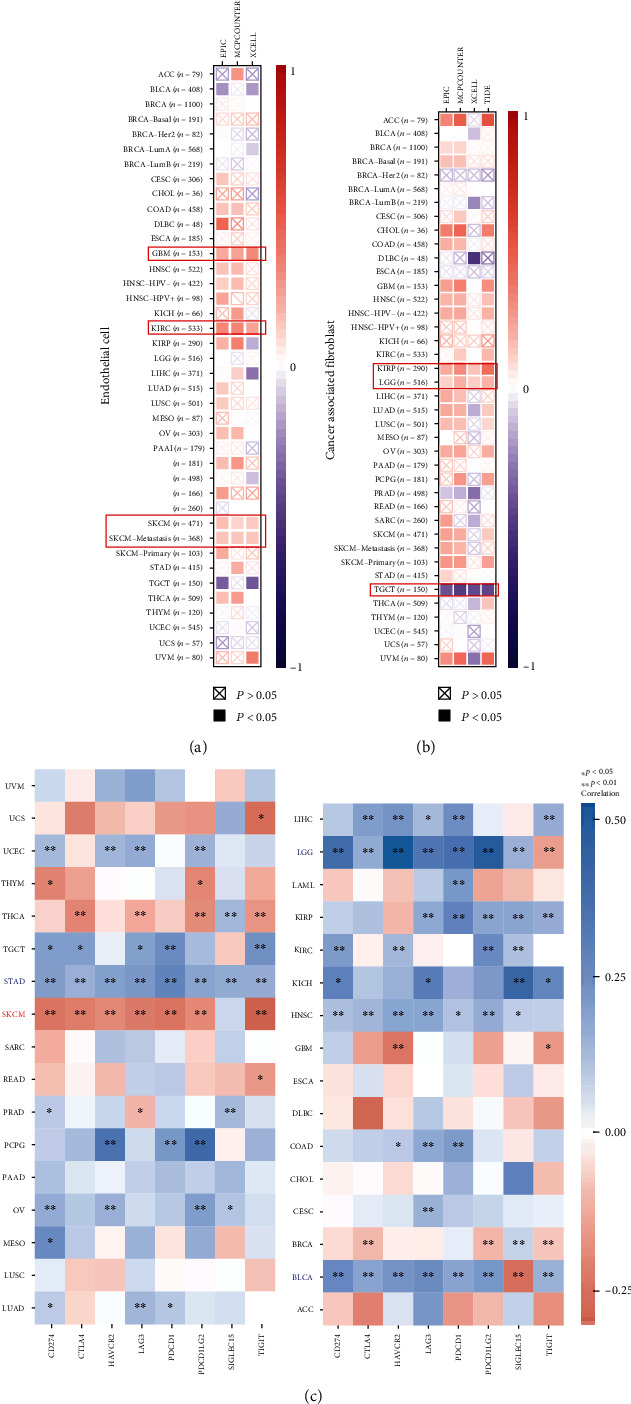
Correlation between the expression level of GRWD1 and endothelial cells, infiltration of cancer-associated fibroblasts, and expression levels of immune checkpoint-related genes. (a, b) The EPIC, MCPcounter, xCell, and TIDE algorithms were used for the correlation analysis of the infiltration levels of cancer-related endothelial cells and fibroblasts and the expression level of GRWD1 in all types of cancer in TCGA. Red indicates a positive correlation (0–1), and blue shows a negative correlation (−1–0). *P* value < 0.05 represents a statistically significant difference. Correlation values that are not statistically significant are marked with crosses. (c) Heat map indicates the expression levels of immune checkpoint-related genes in different tumor tissues; the horizontal axis represents different tumor tissues, the vertical axis shows the expression levels of immune checkpoint-related genes, blue indicates a positive correlation, and red represents a negative correlation. ^∗^*P* < 0.05; ^∗∗^*P* < 0.01.

Cancer-associated fibroblasts are a group of activated fibroblasts with significant heterogeneity and plasticity in the tumor microenvironment. Endothelial cells play essential roles in regulating tumor initiation, progression, and metastasis. The abnormal vascular network formed by endothelial cells in tumors may constitute an immunosuppressive environment and inhibit the infiltration of effector T cells, including CD8+ T cells. The malignant transformation of tumor-related fibroblasts into the tumor microenvironment is closely associated with tumor evolution [18–20]. Therefore, we utilized the TIMER, CIBERSORT, CIBERSORT-ABS, TIDE, xCell, MCPcounter, quanTIseq, and EPIC algorithms in TIMER2 to explore the correlation between different cell infiltration levels and the expression levels of GRWD1 in multiple types of cancer. Importantly, we found that in GBM, KIRC, SKCM, and metastatic SKCM, the expression level of GRWD1 was positively correlated with the estimated infiltration value of endothelial cells. In KIRP and LGG, the expression level of GRWD1 was also positively correlated with infiltration of cancer-associated fibroblasts, while it was negatively correlated with TGCT (Figures 5(a) and 5(b)). Immune checkpoint-related genes have markedly attracted scholars' attention in cancer treatment. Therefore, we explored the correlation between the expression level of GRWD1 and the expression levels of immune checkpoint-related genes through coexpression analysis. We found that the expression level of GRWD1 was positively correlated with the expression levels of immune checkpoint-related genes (CD274, CTLA4, HAVCR2, LAG3, PDCD1, PDCD1LG2, SIGLEC15, and TIGIT) in most types of cancer, especially in STAD. In LGG and BLCA tissues, the expression level of the SIGLEC15 gene was negatively correlated with the expression level of GRWD1. However, in SCKM tissues, the expression levels of 7 immune checkpoint-related genes were negatively correlated with the expression level of GRWD1 (Figure 5(c)).

### 3.7. GRWD1-Related Gene Enrichment Analysis

**Figure 6:**
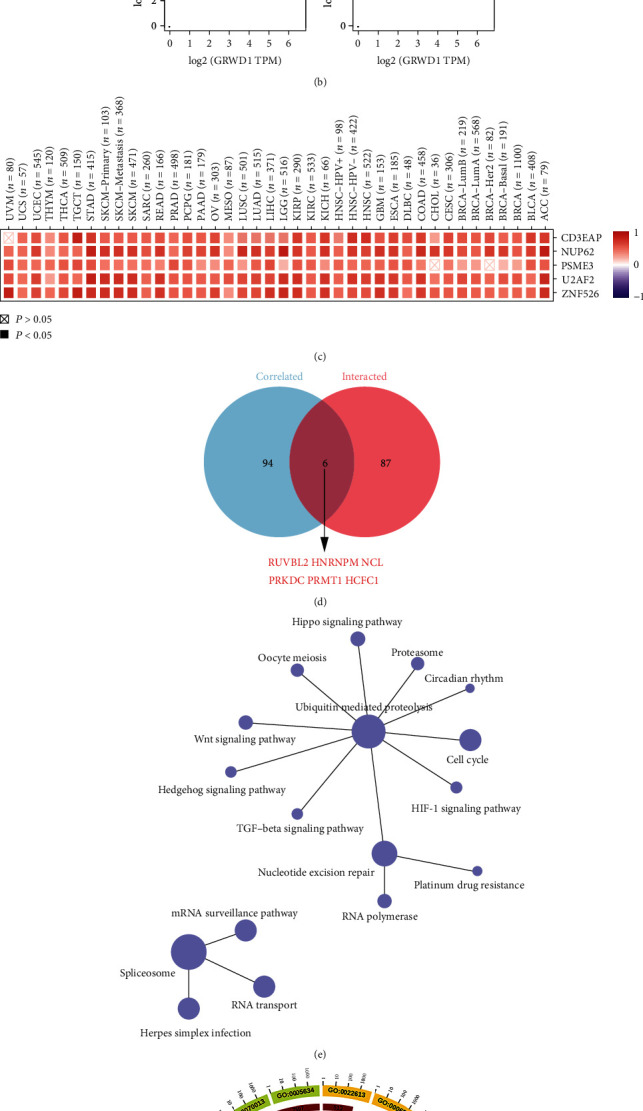
GRWD1-related gene enrichment analysis. (a) The interaction network of the 93 GRWD1-interacting proteins, which were consistent with experimental results from the BioGRID database. (b) Using the GEPIA2, we obtained the top 100 genes with a similar expression pattern to GRWD1 in different types of cancer in TCGA, and we analyzed the correlation between the expression level of GRWD1 and the expression levels of NUP62, CD3EAP, ZNF526, U2AF2, and PSME3. (c) In most types of cancer, the expression level of GRWD1 was strongly positively correlated with the expression levels of these five genes. (d) A Venn diagram displaying the two datasets. (e) The results of KEGG pathway enrichment analysis. (f) The results of GO functional analysis.

GRWD1 can regulate chromatin dynamics and minichromosomal maintenance by promoting chromatin openness. Previous research has not fully revealed its role in tumorigenesis. Therefore, we conducted a series of pathway enrichment analyses on GRWD1-interacting proteins and genes with a similar expression pattern to GRWD1. A total of 93 GRWD1-interacting proteins were identified, which were consistent with experimental results from the BioGRID database.  Figure 6(a) shows the interaction network of these 93 proteins. Then, we used the GEPIA2 to obtain the top 100 genes with a similar expression pattern to GRWD1 in different types of cancer in TCGA. It was noted that the expression level of GRWD1 in all types of cancer in TCGA was positively correlated to the expression levels of nucleoporin 62 (NUP62) (*R* = 0.7), DNA-directed RNA polymerase I subunit RPA34 (CD3EAP) (*R* = 0.66), zinc finger protein 526 (ZNF526) (*R* = 0.65), U2 small nuclear RNA cofactor 2 (U2AF2) (*R* = 0.64), and proteasome activator subunit 3 (PSME3) (*R* = 0.62) genes (Figure 6(b)). The heat map data in Figure 6(c) indicated that in most types of cancer, the expression level of GRWD1 was strongly positively correlated with the expression levels of these five genes. We combined the two sets of genes for the GO and KEGG pathway enrichment analyses. The results of the KEGG pathway enrichment analysis revealed that “ubiquitin mediated proteolysis,” “spliceosome,” and “nucleotide excision repair” were involved in the effect of GRWD1 expression on the tumor pathogenesis (Figure 6(e)). The results of GO functional analysis indicated that the majority of these genes were related to the “binding” of various molecules, including protein binding, heterocyclic compound binding, cyclic compound binding, nuclear acid binding, and mRNA binding (Figure 6(f)).

## 4. Discussion

Previous studies have shown that GRWD1 is a new CDT1-binding protein, possessing histone-binding and nucleosome assembly activities, and it may promote the loading of MCM by maintaining chromatin openness at the replication origins [21, 22]. As a cancer promoter, GRWD1 can regulate the level of RPL11/23 through the ubiquitin-protease system and affect the stability of wild-type P53 [3, 23]. In addition, GRWD1 can directly interact with P53 and negatively regulate P53 transcriptional activities [10]. In non-small cell lung cancer, GRWD1 can activate the cell proliferation and migration abilities via the Notch pathway [11]. The results of phylogenetic tree analysis showed that the structure of the GRWD1 protein was relatively conserved among different species. However, it is highly essential to further concentrate on the expression level of GRWD1 to explore its specific functions in different cellular processes.

Further research is required to indicate whether GRWD1 can play a role in the pathogenesis of different types of cancer through some common molecular mechanisms. Therefore, we analyzed the data of 33 different tumors in TCGA and CPTAC and comprehensively detected the molecular characteristics of the GRWD1 gene and its expression level, as well as genetic alteration, DNA methylation level, and protein phosphorylation level. Our results revealed that GRWD1 was overexpressed in tissues of BLCA, BRCA, CHOL, COAD, ESCA, HNSC, LIHC, LUAD, LUSC, READ, STAD, PRAD, DLBC, GBM, PAAD, TGCT, and THYM. The expression level of GRWD1 in KICH, KIRP, and KIRC tissues showed an opposite trend, and the expression level of GRWD1 was correlated to only the KIRC tumor stage. The difference in the expression level of GRWD1 in different types of cancer may reflect its different functions and mechanisms. Concerning the high expression level of GRWD1 in tumor tissues, the expression level of GRWD1 in only LUAD tissue could predict OS. However, a low expression level of GRWD1 in KIRC tissue was associated with longer OS and RFS, which was consistent with the pathological staging results of GRWD1 and KIRC tumors. These results indicate that the expression level of GRWD1 is more likely to be used as a biomarker to predict the prognosis of KIRC patients.

In addition, the results of the current research, for the first time, revealed that the expression level of GRWD1 in some tumor tissues was correlated to the level of immune infiltration of endothelial cells and cancer-associated fibroblasts. We also found that the expression level of GRWD1 was positively correlated with the expression levels of immune checkpoint-related genes in most types of cancer. As another novelty of the current research, a potential correlation was identified between the expression level of GRWD1 in tumor tissues in TCGA and MSI/TMB. Additionally, we found that the expression level of GRWD1 in KIRC tissue was positively correlated with MSI and the expression level of an immune checkpoint-related gene (CD274). Tumors with a high MSI were shown to have a greater response rate to antiprogrammed cell death 1 (PD-1)/programmed death-ligand 1 (PD-L1) antibody therapy [24]. Our findings may assist clinicians to screen KIRC patients who are eligible for anti-PD-1/PD-L1 antibody therapy.

We also performed a series of pathway enrichment analyses on GRWD1-interacting proteins and genes with a similar expression pattern to GRWD1. We identified the potential roles of “ubiquitin mediated proteolysis,” “spliceosome,” and “binding” of various molecules. It has been reported that GRWD1 can interact with E3 ubiquitin ligase to affect the stability of a ribosomal protein or directly interact with the ribosomal protein to influence the stability of downstream P53 in colorectal cancer cells. GRWD1 can also directly interact with P53. In addition, the results of our study showed that GRWD1 included the mRNA binding protein and nucleic acid-binding protein. This indicates that in addition to influencing DNA replication, GRWD1 could also interact with specific mRNA biological processes. In the next research, we will further analyze the unprecedented performance of GRWD1 in KRIC tissue samples.

According to the data obtained from TCGA, we found that, in addition to a significant negative correlation between probes in the promoter region and the expression level of GRWD1, there was a significant negative correlation between the majority of probes in nonpromoter regions and the expression level of GRWD1. The potential relationship between the DNA methylation level of the GRWD1 gene and pathological features of TGCT deserves further study.

In summary, using pancancer analysis of GRWD1, we found that the expression level of GRWD1 was correlated to the clinical prognosis, protein phosphorylation level, immune cell infiltration, immune checkpoint-related genes, TMB, and MSI in diverse types of cancer. The existence of a statistical correlation can better clarify the role of GRWD1 in tumorigenesis.

## 5. Conclusions

GRWD1 was overexpressed in most types of cancer. The expression level of GRWD1 was significantly correlated to the prognosis of some types of cancer, TMB/MSI, and immune checkpoint-related genes. Therefore, GRWD1 has broad application prospects for the treatment of some types of cancer. The tumor suppression potential of GRWD1 in KRIC and its correlation with MSI and the CD274 gene may be significant for the therapy of KRIC.

## Data Availability

All data was obtained from the public database described in Materials and Methods.

## Supplementary Materials

Supplementary Materials Supplementary Figure 1: structural characteristics of GRWD1 in different species. (A) Location of human GRWD1 gene in hg38; (B) conserved protein domains of GRWD1 in different species. Supplementary Figure 2: the phylogenetic tree of GRWD1. GRWD1 phylogenetic tree displays 20 species based on the NCBI taxonomy browser. Supplementary Figure 3: the expression level of GRWD1 in different tissues and cells under physiological conditions. (A) GRWD1 mRNA expression in different tissues using data obtained from HPA, GTEx, and FANTOM5 databases; (B) GRWD1 mRNA expression in different cells using data obtained from HPA, GTEx, and FANTOM5 databases. Supplementary Figure 4: comparison of the expression level of GRWD1 using the data obtained from the GTEx database. Comparing the expression level of GRWD1 in cancer tissues (LGG, OV, and SKCM) using data obtained from the GTEx database. Supplementary Figure 5: the relationship between the expression level of GRWD1 and pathological staging. There was no significant correlation between the expression level of GRWD1 and pathological staging. Supplementary Figure 6: the correlation between the expression level of GRWD1 and tumor prognosis was assessed using the Kaplan-Meier plotter. Kaplan-Meier plotter was used for survival analysis of (A) breast cancer, (B) liver cancer, (C) gastric cancer, (D) lung cancer, and (E) ovarian cancer (OS: overall survival; DMFS: distant metastasis-free survival; RFS: relapse-free survival; PFS: progression-free survival; PPS: postprogression survival; FP: first progression; DSS: disease-free survival). Supplementary Figure 7: the relationship between the expression level of GRWD1 and tumor mutation burden. Spearman's correlation analysis of TMB and the expression level of GRWD1. The horizontal axis represents the correlation coefficient between genes and TMB, the vertical axis indicates different tumors, the size of the dots represents the correlation coefficients, and different colors indicate *P* values. Supplementary Figure 8: the relationship between the expression level of GRWD1 and MSI. Spearman's correlation analysis of MSI and the expression level of GRWD1. The horizontal axis represents the correlation coefficient between genes and MSI, the vertical axis indicates different tumors, the size of the dots represents the correlation coefficients, and different colors show *P* values. Supplementary Figure 9: the relationship between DNA methylation level of the GRWD1 gene and pathological features of TGCT. (A) Data obtained from UALCAN showed that the DNA methylation level of the GRWD1 increased in promoter regions. (B) DNA methylation level of GRWD1 was determined using data obtained from MEXPRESS.
