# Supplementary material for: Comprehensive Analysis of Glutamate-Rich WD Repeat-Containing Protein 1 and Its Potential Clinical Significance for Pancancer
Source: Biomed Res Int. 2021 Sep 27;2021:8201377. doi: 10.1155/2021/8201377 (PMC8490071; doi:10.1155/2021/8201377)
Supplement: Supplementary Materials — Supplementary Figure 1: structural characteristics of GRWD1 in different species. (A) Location of human GRWD1 gene in hg38; (B) conserved protein domains of GRWD1 in different species. Supplementary Figure 2: the phylogenetic tree of GRWD1. GRWD1 phylogenetic tree displays 20 species based on the NCBI taxonomy browser. Supplementary Figure 3: the expression level of GRWD1 in different tissues and cells under physiological conditions. (A) GRWD1 mRNA expression in different tissues using data obtained from HPA, GTEx, and FANTOM5 databases; (B) GRWD1 mRNA expression in different cells using data obtained from HPA, GTEx, and FANTOM5 databases. Supplementary Figure 4: comparison of the expression level of GRWD1 using the data obtained from the GTEx database. Comparing the expression level of GRWD1 in cancer tissues (LGG, OV, and SKCM) using data obtained from the GTEx database. Supplementary Figure 5: the relationship between the expression level of GRWD1 and pathological staging. There was no significant correlation between the expression level of GRWD1 and pathological staging. Supplementary Figure 6: the correlation between the expression level of GRWD1 and tumor prognosis was assessed using the Kaplan-Meier plotter. Kaplan-Meier plotter was used for survival analysis of (A) breast cancer, (B) liver cancer, (C) gastric cancer, (D) lung cancer, and (E) ovarian cancer (OS: overall survival; DMFS: distant metastasis-free survival; RFS: relapse-free survival; PFS: progression-free survival; PPS: postprogression survival; FP: first progression; DSS: disease-free survival). Supplementary Figure 7: the relationship between the expression level of GRWD1 and tumor mutation burden. Spearman's correlation analysis of TMB and the expression level of GRWD1. The horizontal axis represents the correlation coefficient between genes and TMB, the vertical axis indicates different tumors, the size of the dots represents the correlation coefficients, and different colors indicate P value [file 8201377.f1.docx]

**Supplementary Materials**


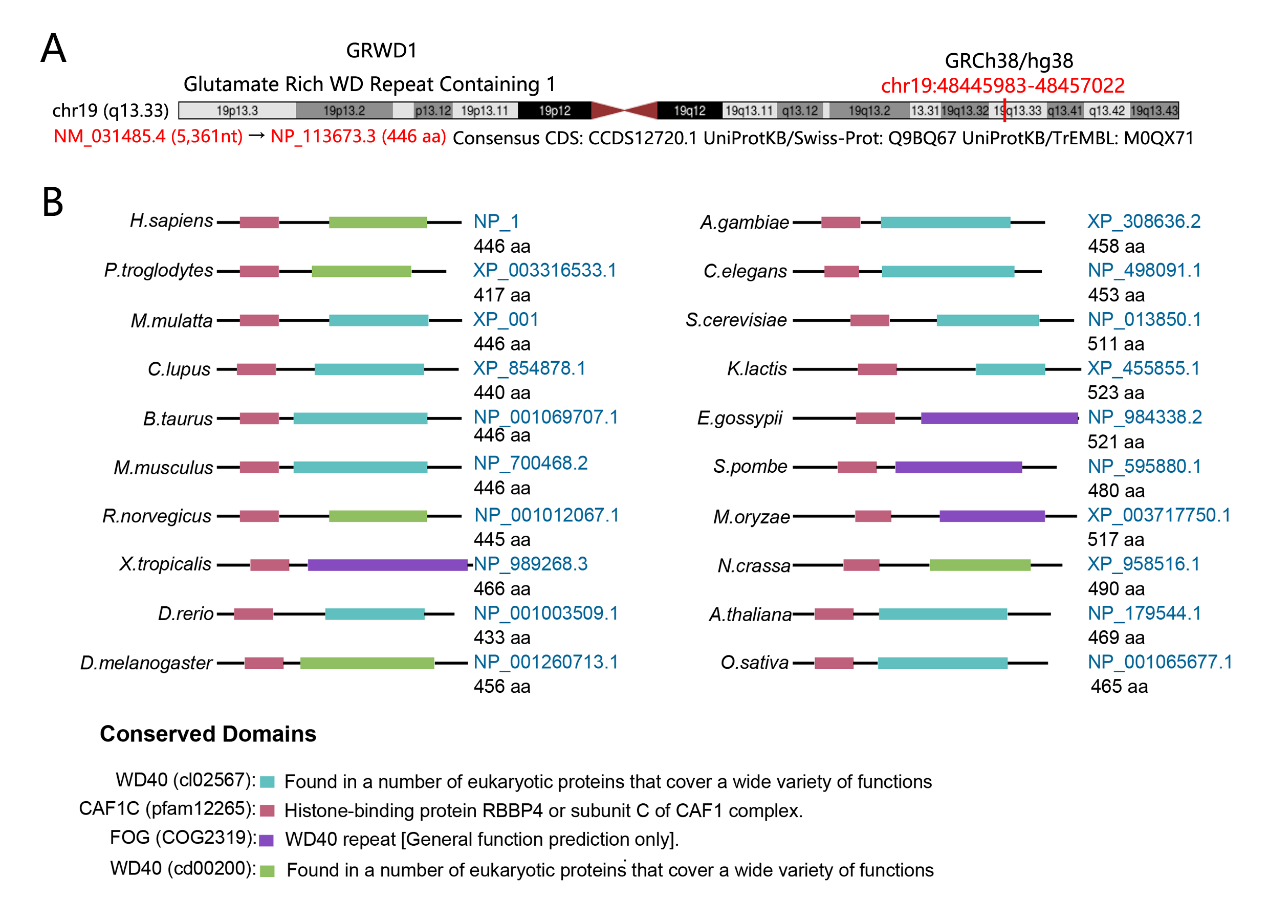
**Supplementary Figure 1: Structural characteristics of GRWD1 in different species.** (A) Location of human GRWD1 gene in hg38; (B) Conserved protein domains of GRWD1 in different species.


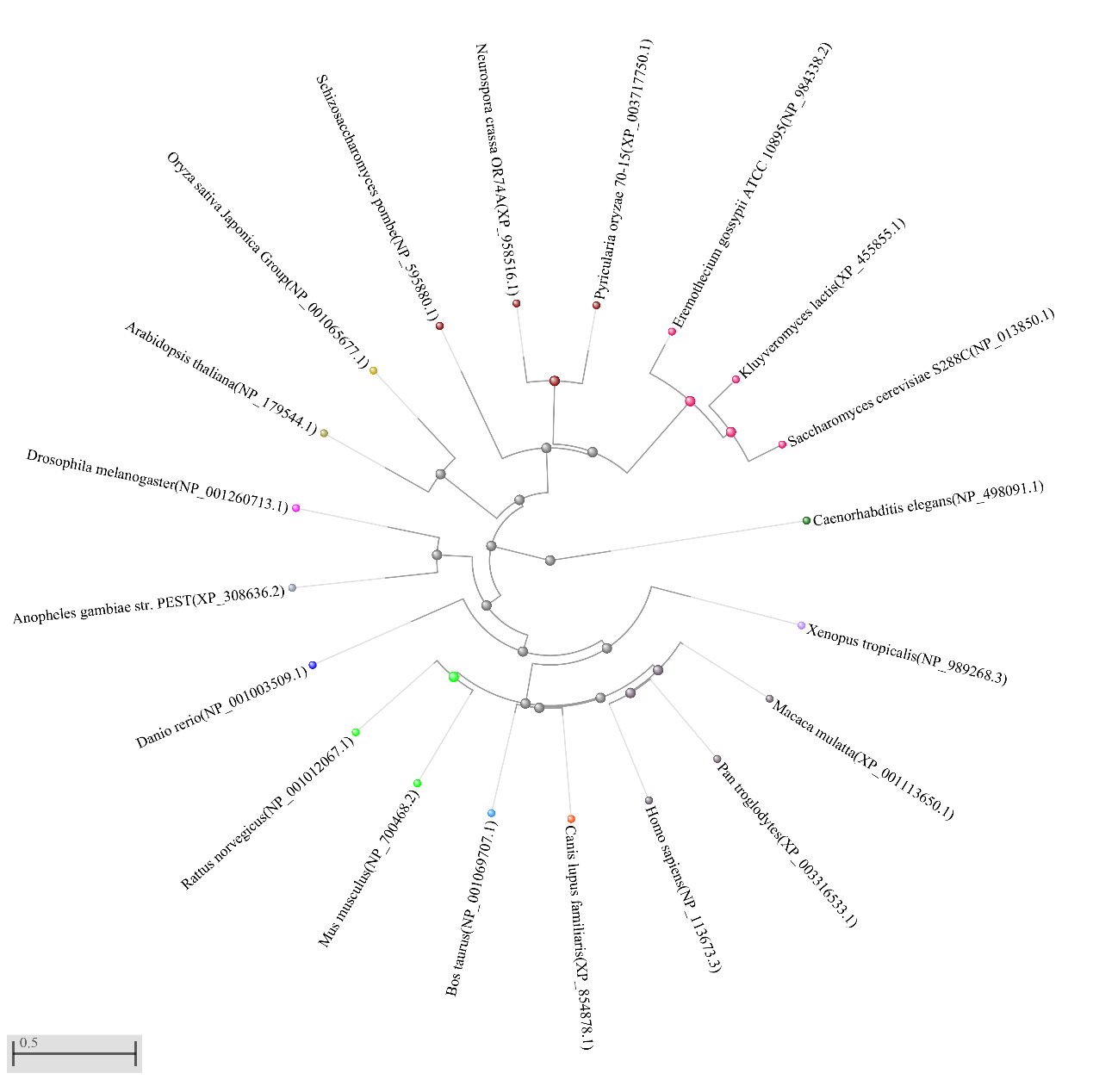


**Supplementary Figure 2: The phylogenetic tree of GRWD1.** GRWD1 phylogenetic tree displays 20 species based on the NCBI taxonomy browser.


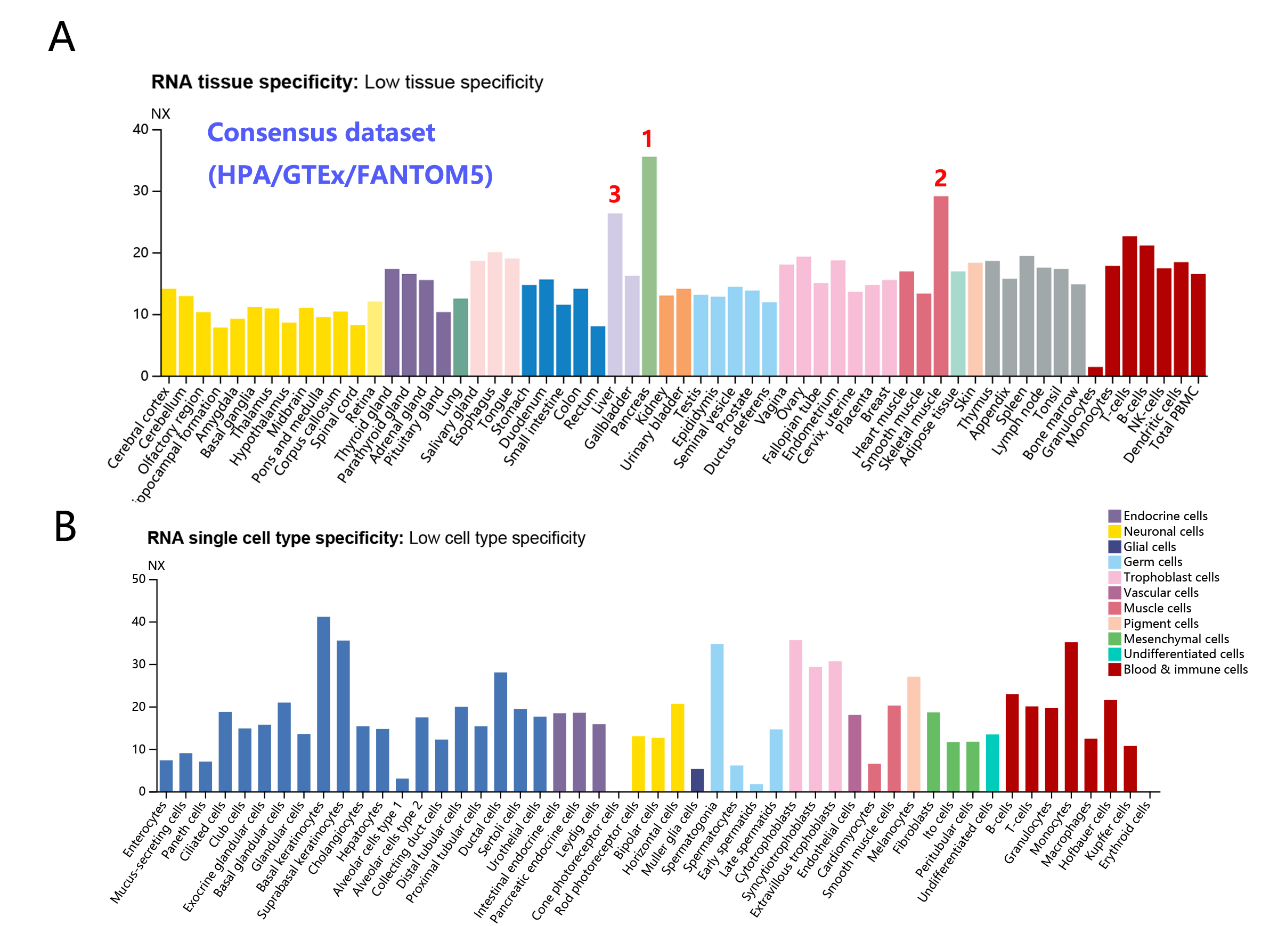


**Supplementary Figure 3: The expression level of GRWD1 in different tissues and cells under physiological conditions.** (A) GRWD1 mRNA expression in different tissues using data obtained from HPA, GTEx, and Fantom5 databases; (B) GRWD1 mRNA expression in different cells using data obtained from HPA, GTEx, and Fantom5 databases.


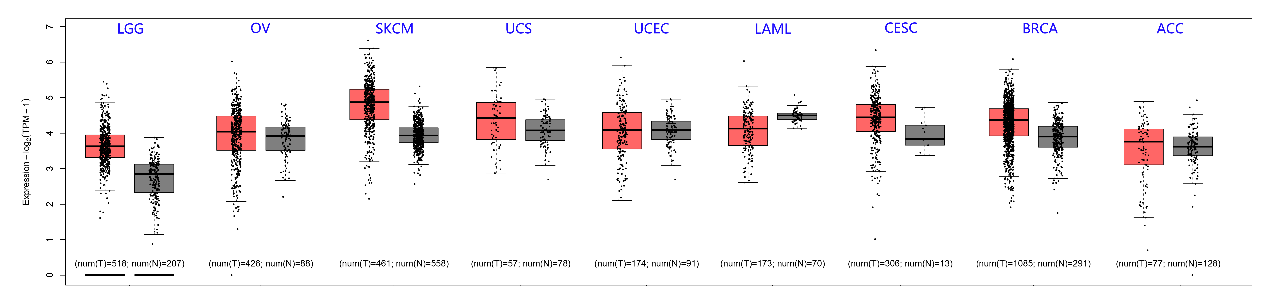
**Supplementary Figure 4: Comparison of the expression level of GRWD1 using the data obtained from GTEx database.** Comparing the expression level of GRWD1 in cancer tissues (LGG, OV, and SKCM) using data obtained from the GTEx database.


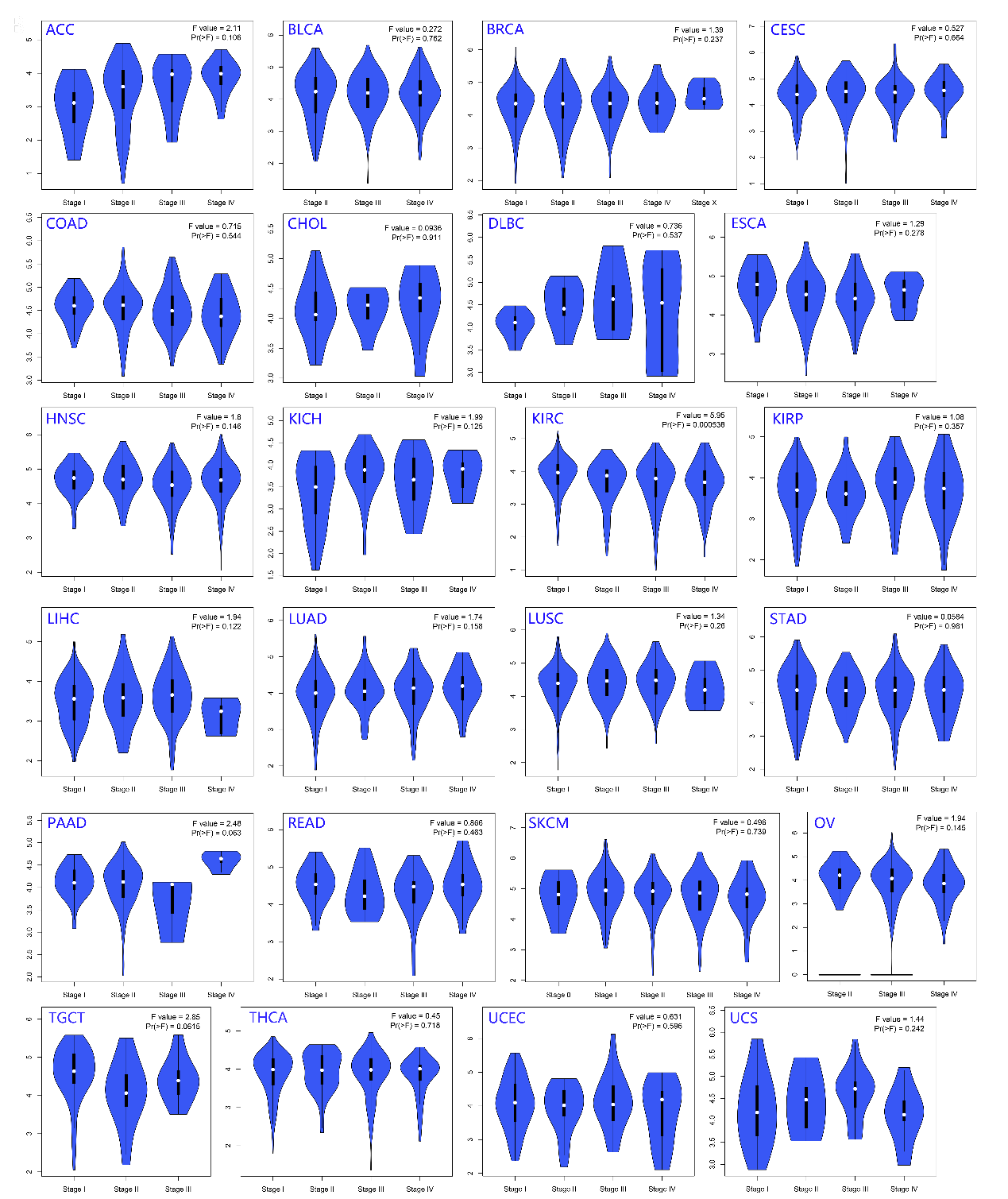


**Supplementary Figure 5: The relationship between the expression level of GRWD1 and pathological staging.** There was no significant correlation between the expression level of GRWD1 and pathological staging.


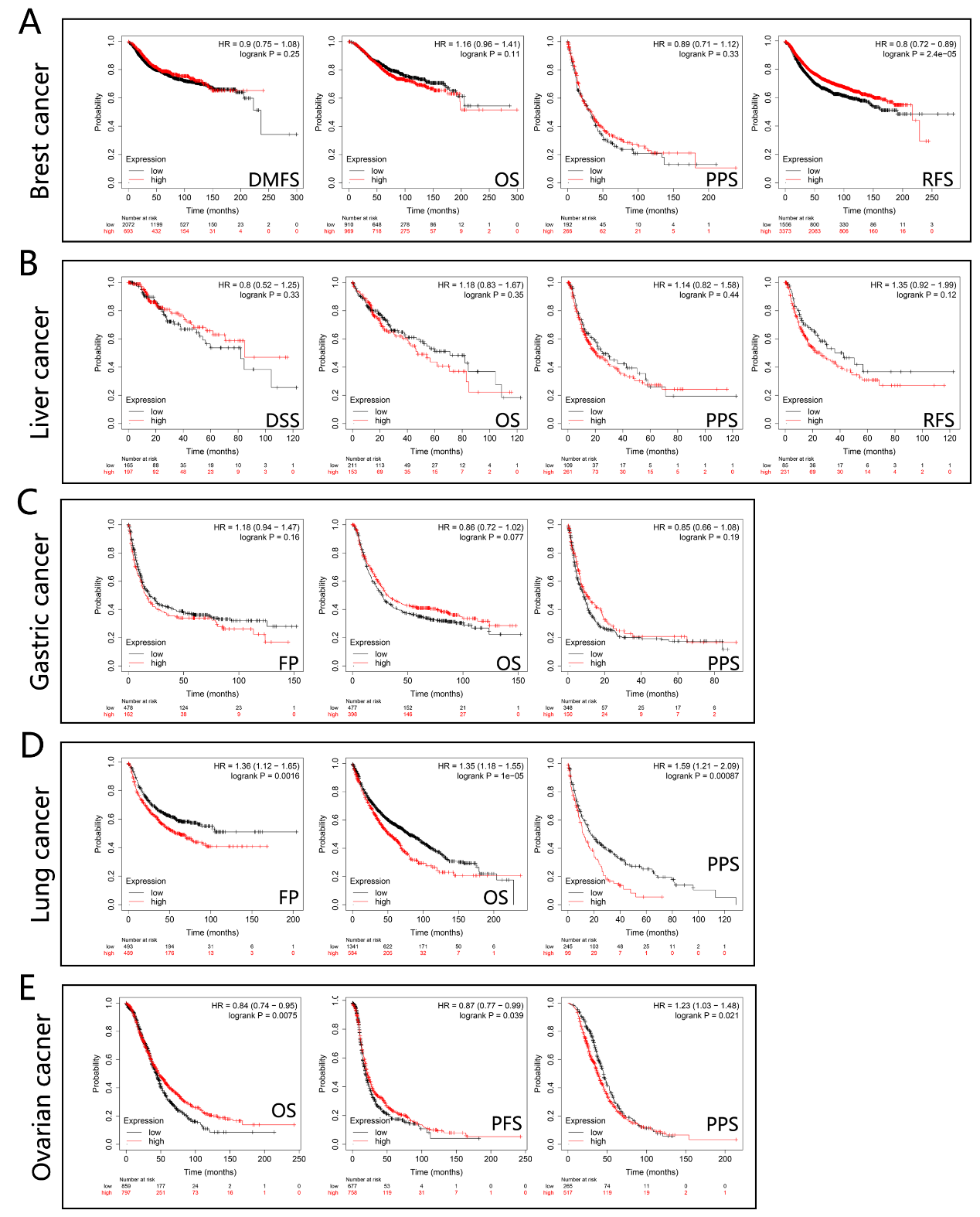


**Supplementary Figure 6: The effect of GRWD1 gene expression on tumor prognosis was studied with the Kaplan-Meier plotter.** Kaplan-Meier plotter is used for **(A)** breast cancer, **(B)** liver cancer, **(C)** gastric cancer, **(D)** lung cancer and **(E)** ovarian cancer survival analysis. (OS: overall survival; DMFS: distant recurrence-free survival; RFS: relapse-free survival; PFS: progression free survival; PPS: post progression survival; FP: first progression; DSS: disease free survival)


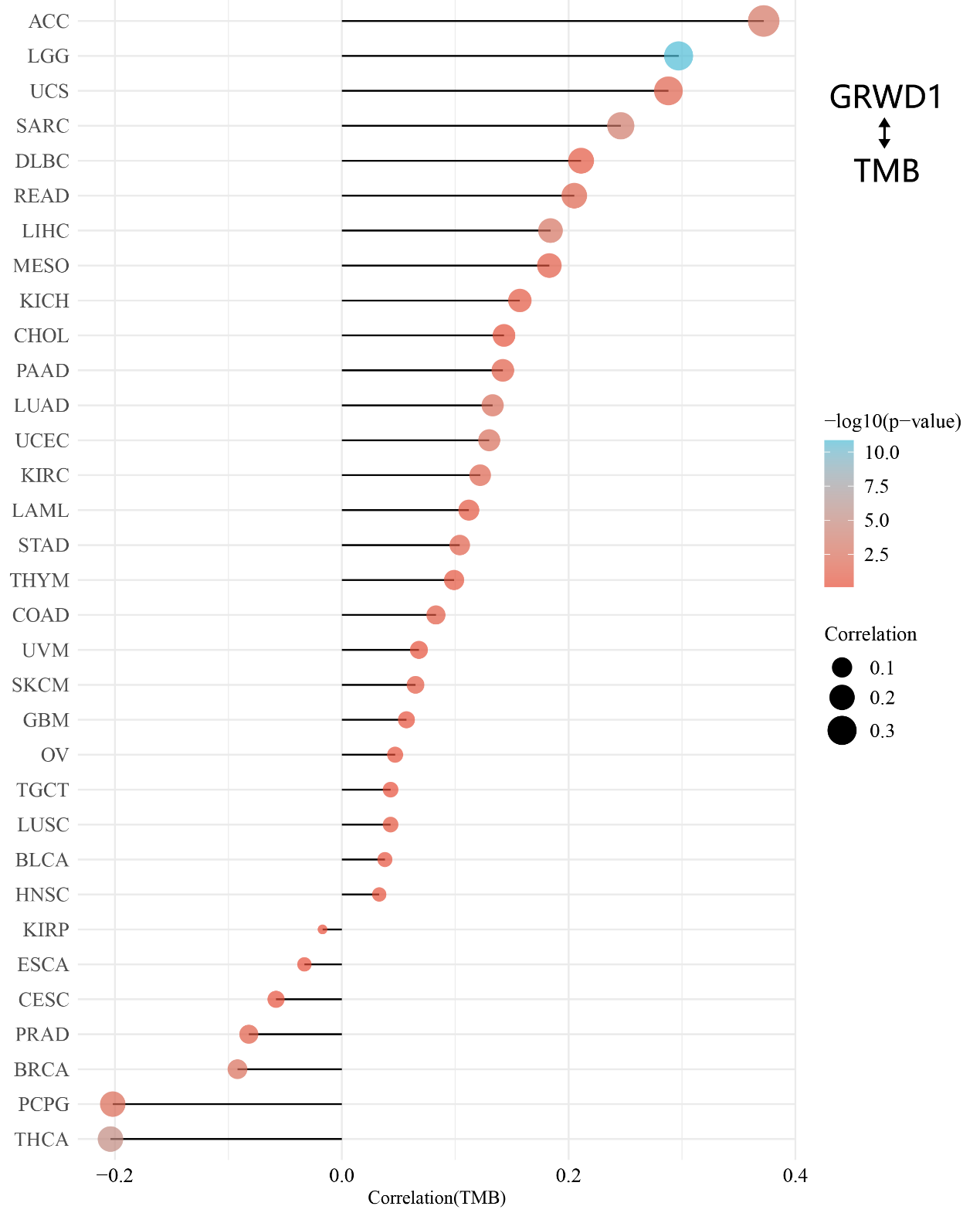
**Supplementary Figure 7: The relationship between the expression level of GRWD1 and tumor mutation burden.** The Spearman’s correlation analysis of TMB and the expression level of GRWD1. The horizontal axis represents the correlation coefficient between genes and TMB, the vertical axis indicates different tumors, the size of the dots represents the correlation coefficients, and different colors indicate *P*-values.


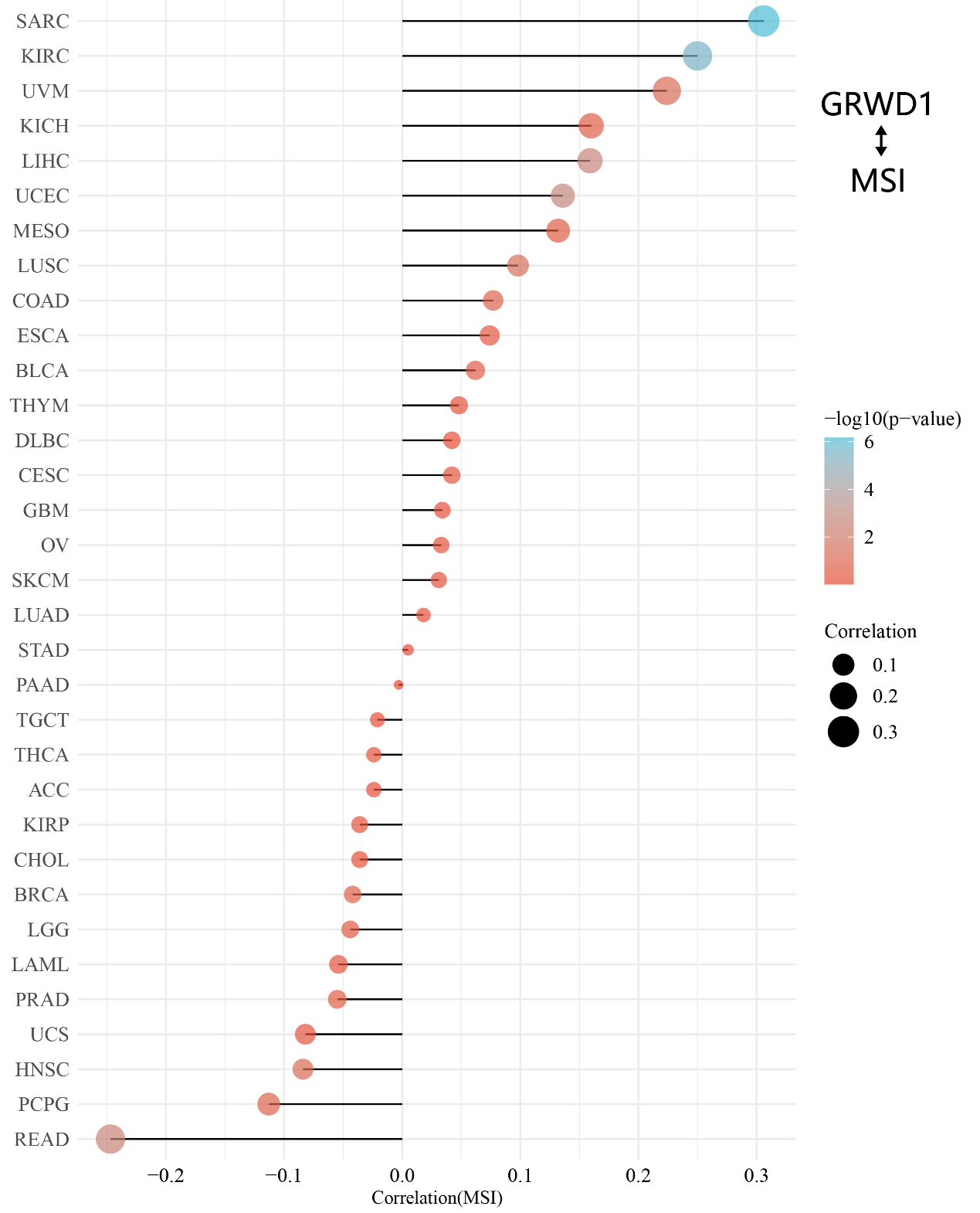


**Supplementary Figure 8: The relationship between the expression level of GRWD1 and MSI.** The Spearman’s correlation analysis of MSI and the expression level of GRWD1. The horizontal axis represents the correlation coefficient between genes and MSI, the vertical axis indicates different tumors, the size of the dots represents the correlation coefficients, and different colors show *P*-values.


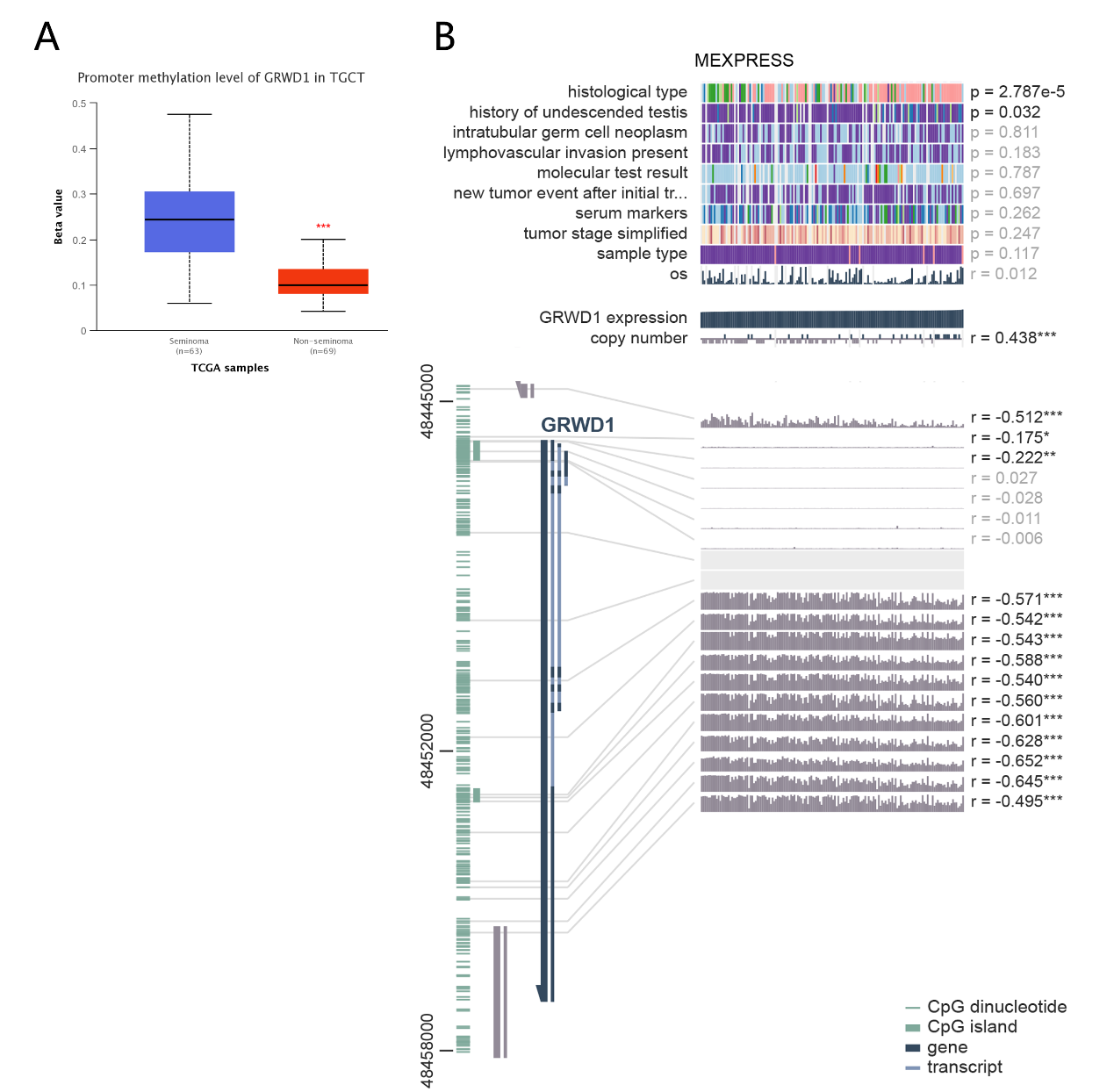


**Supplementary Figure 9: The relationship between DNA methylation level of GRWD1 gene and pathological features of TGCT.** (A) Data obtained from UALCAN showed that the DNA methylation level of the GRWD1 increased in promoter regions. (B) DNA methylation level of GRWD1 was determined using data obtained from MEXPRESS.
